# Prevalence of vitamin D deficiency and its association with metabolic derangements among children with obesity

**DOI:** 10.1186/s12887-019-1558-8

**Published:** 2019-06-08

**Authors:** S. G. S. Adikaram, D. B. D. L. Samaranayake, N. Atapattu, K. M. D. L. D. Kendaragama, J. T. N. Senevirathne, V. Pujitha Wickramasinghe

**Affiliations:** 1 Colombo South Teaching Hospital, Kalubowila Colombo, Sri Lanka; 2 Department of Community Medicine, Faculty of Medicine, University of Colombo, Colombo, Sri Lanka; 3 Lady Ridgeway Hospital, Colombo, Sri Lanka; 4 Department of Paediatrics, Faculty of Medicine, University of Colombo, Kynsey Road, Colombo, Sri Lanka

**Keywords:** Vitamin D deficiency, Sri Lankan children, Childhood obesity

## Abstract

**Background:**

It is known that obesity is associated with vitamin D deficiency and observational studies have shown vitamin D deficiency to be linked with the development of type 2 diabetes. There are no comprehensive data regarding vitamin D deficiency in children with obesity in Sri Lanka and the objective of the study was to assess the prevalence of Vitamin D deficiency and its association with metabolic derangements among children with obesity.

**Methodology:**

Two hundred and two children between 5 and 15 years of age attending the obesity clinic Lady Ridgeway Hospital (LRH) were recruited excluding those having possible secondary causes for obesity. Blood was drawn after 12-h overnight fast for fasting blood glucose(FBG), lipid profile, serum insulin, alanine aminotransferase (ALT),aspartate aminotransferase(AST), Vitamin D, parathyroid hormone(PTH),high sensitivity C reactive protein(hs-CRP). Oral glucose tolerance test (OGTT) was done with 2 h random blood glucose. Anthropometry, blood pressure were measured, and body fat mass was assessed using bio-impedance.

**Results:**

Vitamin D deficiency (< 20 ng/ml) was seen in 152(75.2%) children and 43(21.3%) had insufficient (20-30 ng/ml) levels. Skin fold thickness, fasting and post-glucose insulin, HOMA-IR, PTH, LDL, Serum cholesterol and hs-CRP showed statistically significant negative correlations with Vitamin D levels.

**Conclusions:**

Vitamin D deficiency was significantly high in Sri Lankan children with obesity and showed significant negative correlations with indicators of insulin resistance and adiposity.

## Background

Prevalence of obesity in children has risen remarkably worldwide. Despite a known genetic contribution, the increase in paediatric obesity has been attributed mainly to diet and sedentary lifestyle. Obesity is one of the most important modifiable risk factors for the prevention of number of chronic diseases. The resulting socio-economic and public health burden due to its consequences (development of hypertension, diabetes mellitus, social discrimination etc.) is growing steeply. Although obese individuals are thought to be adequately nourished, micronutrient deficiencies have been identified to be prevalent possibly due to the large fat mass acting as a reservoir for fat-soluble vitamins and nutrients or due to consumption of food rich in calories but poor in other nutrients.

Vitamin D is a fat-soluble vitamin with a half-life of 4–6 weeks and its deficiency was observed in obese individuals in several studies although the exact reason is not known. Several possibilities have been suggested to explain the lower 25(OH)D levels observed in children with obesity, including decreased sun exposure due to sedentary lifestyle, poor diet(skipping breakfast, increased soda intake, and increased juice intake) and increased clearance of 25(OH)D due to storage in adipose tissue [1] .

Many observational studies have investigated the relationship between vitamin D statuses and type 2diabetes mellitus (T2DM).Apart from low vitamin D levels being associated with low bone mineral density due to its role in calcium homeostasis, it also plays a crucial role in insulin secretion and maintaining glucose homeostasis via its endocrine mechanisms [2–4]. Pancreatic cells are known to contain vitamin D receptors and vitamin D binding proteins and calcium play a role in insulin secretion [3, 5].

Other possible explanation for vitamin D deficiency contributing to the development of T2DM is the possible anti-inflammatory role. Since the immunomodulatory functions of vitamin D are incontestable, its deficiency in obesity may coincide with enhanced systemic inflammation. It has also been shown that low-grade inflammation is associated with reduced insulin sensitivity. This explains the place for measuring high sensitivity C reactive protein (hs-CRP) in detecting impaired insulin sensitivity and the development of metabolic syndrome and T2DM.These possible anti-inflammatory properties of vitamin D is supported by the results of a recently published paediatric study showing an association between low vitamin D level and increased systemic inflammation. It is also postulated that it may be contributing to activation of pro-inflammatory, pro-diabetic and atherogenic pathways in children with obesity [6].

There are no comprehensive data on prevalence of vitamin D deficiency among children in Sri Lanka. However, few studies have shown the prevalence of vitamin D deficiency among pre-school children. Vitamin D deficiency using a higher cut off value (< 35 nmol/l) was seen in 26% of male and 25% of female children in the southern province of Sri Lanka [7]. Similar study done recently showed vitamin D deficiency (< 10 ng/ml/l)in 5.6% and insufficiency(10-20 ng/ml) in 29.1% of a group of children from an urban area in western province of Sri Lanka [8].In the light of the increasing incidence of childhood obesity in Sri Lanka and significantly higher prevalence of vitamin D deficiency in the childhood population, we assessed the prevalence of vitamin D deficiency and its association with obesity related metabolic derangements among a group of obese Sri Lankan children attending a tertiary care hospital.

## Methodology

### Study population

This was a cross sectional study where 202 children, having a body mass index (BMI)more than 2 standard deviation score (SDS) above the median for age and sex according to World Health Organization standards [9], attending the obesity clinic at Lady Ridgeway Hospital for Children, Colombo were consecutively recruited after obtaining informed written consent. Children with obesity due to genetic causes (e.g.Prader Willi syndrome etc.), endocrine pathology (e.g. hypothyroidism, Cushing syndrome etc.), those who are on long term medication and those who didn’t consent were excluded. Relevant demographic and clinical information were collected using an interviewer-administered questionnaire.

The Ethics Review Committee of Faculty of Medicine, University of Colombo, approved the study protocol (EC-15-010). All the participants were recruited after obtaining informed written consent from either of the parents or the guardian. In addition, assent was obtained from children above 12 years of age.

### Data collection

Height was measured according to the standard protocols and the weight was measured using an electrical weighing scale, which was calibrated regularly. BMI was calculated as weight (kg) divided by height (m) squared. The waist circumference (WC) was measured horizontally at the midpoint betweenthe lower point of costal margin and highest point of iliac crest in mid axillary line. Body fat was measured by bioelectrical impedance analysis (BIA) technique using InBody 230® (InBodyInc, South Korea). Skin fold thicknesses of 4 sites (triceps, biceps, subscapular, and supra iliac) was measured (Harpendens Caliper®, UK) using standard protocol. Central skin fold thickness was calculated by the summation of supra iliac and subscapular values.

After 12 h overnight fast, blood was taken for fasting blood glucose (FBG), serum insulin, vitamin D level, alanine aminotransferase (ALT),aspartate aminotransferase(AST), lipid profile, serum parathyroid hormone(PTH),high sensitivity C reactive protein(hs-CRP) and serum creatinine. Oral glucose tolerance test (OGTT) was performed using 1.75 g of anhydrous glucose (maximum 75 g of anhydrous glucose) and blood was taken for random blood glucose (RBG) and serum insulin after 2 h of oral glucose load. Within one hour of collection, blood was centrifuged to separate serum and stored in aliquots at -20 °C until analysis.

Ultra sound scan (USS) of the abdomen was conducted to assess the degree of fat deposition in the liver.

### Follow up

Participants were followed up at the obesity clinic and the results were discussed on an individual basis and required interventions were made.

### Analysis

Serum and plasma separation was carried out by centrifugation of samples at 2500 rpm within one hour of collection. Both serum and plasma were separated into small aliquots of 2 ml and stored at -20^0^C in eppendorf tubes until analysis. Samples for Blood sugar estimation were analyzed within 2 h of collection. Analysis of serum samples for clinical chemistry was carried out using Dimension ready to use reagent packs on Dimension Clinical Chemistry system (Dimension _X_^pand^ Plus Random access automated clinical chemistry analyzer, Semens Healthcare Diagnostics Inc. USA).

FBG and RBG assessment was done using the Hexokinase method [10]. Serum Insulin, PTH and vitamin D assays were carried out using immuno assays. Serum Insulin and PTH tests were done on fully automated random access IMMULITE® 1000 immuno assay system (Semens Healthcare Diagnostics Inc. USA)and assessed by solid-phase, two-site chemiluminescent enzyme-labelledimmunometric assay [11].

Assessment of serum Vitamin D was done by LIASON 25OH vitamin D TOTAL assay using chemiluminescent immunoassay technology.

Serum total cholesterol and triglyceride were measured by CHOD- PAP- method (Enzymatic colorimetric test for cholesterol with lipid clearing factor), using reagent kits. Serum HDL-Cholesterol was measured by the method of precipitant and standard for use with cholesterol liquicolor, using commercially available reagent kit. Serum LDL-C concentration was calculated from the total cholesterol concentration, the HDL cholesterol concentration and the triglycerides concentration using standard equation.

(LDL-Cholesterol = Total Cholesterol – HDL-Cholesterol – Triglycerides/5)

Liver enzymes, ALT and AST, were measured by colorimetric method using commercially available kit.hs-CRP was measured by dimension cardio-phase high sensitive colorimetric immunoassay and serum creatinine was measured using Kinetic Jaffe reaction.

### Definitions of cut-off values

Vitamin D deficiency,where 25(OH) D < 20 ng/ml and Vitamin D insufficiency, when 25(OH) D level is between 20 and 29 ng/ml [12].Fasting insulin > 12microIU/ml [13] and 2 h > 75microIU/ml [14].

Dysglycaemia was defined if one of the following were present. Impaired fasting glucose (FBG -100 – 125 mg/dl) or impaired glucose tolerance (OGTT 2-h RBG value 140- 200 mg/dl) or overt diabetes mellitus (FBG > 126 mg/dl or OGTT 2 h RBG > 200 mg/dl). HOMA-IR was taken as elevated when the levels were above 2.5 [15].Cut-off values for serum triglycerides > 150 mg/dl, serum HDL < 40 mg/dl [16], serum cholesterol > 200 mg/dl [17], Serum LDL-C > 130 mg/dl, AST and ALT > 40 IU/l, Serum PTH > 65 pg/ml, hs-CRP > 3 mg/l.

Above + 2 SDS for Systolic and Diastolic BP [18] and WC [19] of relevant references were used as cutoff values. A percentage fat mass > 28.6 in boys and > 33.7% in girls were considered as high [20].

Insulin resistance was assessed using Homeostasis Model Assessment of Insulin Resistance (HOMA-IR) and, was calculated using the following formula: HOMA-IR = [(fasting insulin in μU/ml) × (fasting glucose in mg/dl)]/405 [21].

### Statistical analysis

All the statistical analyses were performed using Statistical Package for the Social Sciences (SPSS). Prevalence of Vitamin D deficiency and its distribution according to the socio-demographic characteristics (age, gender, and ethnicity) of the sample was analyzed.Metabolic characteristics irrespective of the vitamin D levels were analyzed initially. The associations between vitamin D deficiency and metabolic derangements were analyzed and the significance was calculated using chi square test. Correlations between vitamin D levels with anthropometric measures, body fat mass and metabolic parameterswere calculated using Pearson’s correlation coefficients. Significance was considered as *p* < 0.05.

## Results

A total of 202 (males − 70.79%) children were included in the analysis. Mean age was 10.11 years with a SD of 2.1 years. Sixty-eight (48.2%) boys and 23 (39%) girls had entered puberty (Table 1).

**Table 1:** Socio-demographic Characteristics of the sample (*n* = 202)

Socio-demographic characteristics		Male (*n* = 143)	Female (*n* = 59)
		Number (%)	Number (%)
Age	5–8 years	29 (20.3)	10 (16.9)
	8-12 years	84 (58.7)	42 (71.2)
	> 12 years	30 (21.0)	7 (11.9)
Ethnicity	Sinhalese	115 (80.4)	34 (57.6)
	Muslim	16 (11.2)	19 (32.2)
	Tamil	12 (8.4)	6 (10.2)
Pubertal Status^a^	Pubertal	68 (48.2)	23 (39.0)
	Pre-pubertal	73 (51.8)	36 (61.0)
Total	143	143 (70.8)	59 (29.2)

^a^Pubertal state was not recorded in 2 males due to practical difficulties

WC was high in 193 (96.5%) and fat mass was high in 196 (99%) of the sample. Percentage fat mas was high in 197 (99.5%), the mean being 42.53%.Systolic blood pressure was normal in all and diastolic blood pressure was high only in 8 (4%) children. Majority had normal fasting blood glucose (FBG) and 2-h blood glucose values in oral glucose tolerance (OGTT) while FBG was high in 22 (10.9%) children and 2 h blood glucose was high in 23 (11.4%) children. Fasting insulin was high in 106 (52.5%) children and HOMA-IR was high in 107 (53%) children. High densitylipoproteins (HDL) were low in 110 (54.5%) children in contrast to other lipid fractions being normal in majority. Serum cholesterol was high only in 55 (27.2%) and the mean was 184.0 mg/dl. Serum LDL was high in 72 (35.6%), the mean being 121.7 mg/dl. Serum triglyceride was high in 37 (18.3%) and the mean was 113.2 mg/dl. Serum ALT was high in 44 (21.8%) and AST was high in 50 (24.9%) children. High sensitivity CRP had a mean of 5.04 which was above normal. However, 99 (51%) had normal values and 95 (49%)had high values. Serum PTH was normal in majority and only 11 (5.4%) had high values and the mean was 34.9 pg/ml. Distribution of selected anthropometric and metabolic characteristics are shown in Table 2.

**Table 2:** Anthropometric and Metabolic characteristics of the sample (*n* = 202)

Anthropometric / Metabolic Characteristics	Mean (SD)	Normal *N* (%)	High/Abnormal *N* (%)
BMI SD Score	2.75 (0.658)	0	202 (100%)
Waist Circumference SD Score	3.00 (0.598)	7 (3.5)	193 (96.5)
Percentage Fat Mass	42.5 (5.08)	1 (0.5)	197 (99.5)
Insulin Fasting (microIU/ml)	14.0 (9.49)	96 (47.5)	106 (52.5)
Insulin 2 h (microIU/ml)	100.0 (81.4)	101 (50.2)	100 (49.8)
HOMA IR	1.53 (0.500)	95 (47.0)	107 (53.0)
High sensitivity C reactive protein (mg/l)	5.04 (14.658)	99 (51.0)	95 (49.0)

Waist circumference SD score and Percentage fat mass values were not recorded in two and four children respectively due to practical difficulties. 2 h Insulin was not analyzed in *n* = 1 and hs-CRP was not analyzed in *n* = 8 due to sample inadequacy.

Vitamin D analysis showed that most of the children (*n* = 152, 75.2%) had levels lower than 20 ng/ml (deficient range) and 43 (21.3%) had insufficient (21–30 ng/ml) levels. Only 7(3.5%) had normal values ranging from 30.5 ng/ml to 39.3 ng/ml, which were close to the lower normal range. Mean Vitamin D level of the study sample was 17.4 ng/ml (SD - 5.6). The minimum value reported was 6.7 ng/ml and the highest was 39.3 ng/ml.

Distribution of vitamin D deficiency state did not show clear association with socio-demographic characteristics (age, gender and ethnicity). The correlations of Vitamin D levels with selected anthropometric measures and biochemical parameters were assessed and all except HDL showed negative correlations while HDL was positively correlated. The following had statistically significant negative correlations - subscapular SFT, biceps SFT, supra iliac SFT, central SFT, Fasting and 2-h Insulin, HOMA-IR, LDL-C, Serum cholesterol, hs-CRP and PTH (Table 3).

**Table 3:** Correlations of Vitamin D levels with selected anthropometric and metabolic characteristics (*n* = 202)

Anthropometric /Metabolic Characteristics	Correlation Coefficient	Significance
BMI Z score	−0.041	0.541
SFT Triceps	- 0.084	0.236
SFT Biceps	**- 0.147**	**0.037**
SFT Subscapular	**- 0.215**	**0.002**
SFT Supra iliac	**- 0.206**	**0.003**
Central SFT	**−0.229**	**0.001**
Peripheral SFT	−0.135	0.056
Waist Circumference (cm)	- 0.032	0.655
Percentage Fat Mass	−0.034	0.632
Serum Calcium	−0.006	0.928
Parathyroid Hormone (pg/ml)	**− 0.228**	**0.001**
Alkaline phosphatase	−0.131	0.064
Fasting Blood Sugar (mg/dl)	−0.127	0.072
2 h OGTT (mg/dl)	−0.124	0.079
Fasting Insulin (micro IU/ml)	**−0.147**	**0.037**
2 h Insulin (micro IU/ML)	**−0.157**	**0.026**
HOMA IR	**−0.155**	**0.028**
Serum Triglycerides (mg/dl)	−0.087	0.221
High Density Lipoproteins (mg/dl)	0.093	0.187
Low Density Lipoproteins (mg/dl)	**−0.181**	**0.010**
Serum Cholesterol (mg/dl)	**−0.148**	**0.035**
Aspartate aminotransferase (IU/l)	−0.069	0.328
Alanine aminotransferase (IU/l)	−0.075	0.287
High sensitivity C reactive Protein (mg/l)	**−0.142**	**0.048**

*SFT* Skin Fold Thickness, *HOMA IR* Homeostatic Model Assessment of Insulin Resistance, *PTH* Parathyroid hormone, *ALP* Alkaline phosphatase, *HOMA IR* Homeostatic Model Assessment of Insulin Resistance

Data in bold represents *p*-value <0.05

The association between vitamin D deficiency and anthropometric and metabolic abnormalities were assessed and the significance of these associations was tested using chi square test. We could not observe any significant associations with vitamin D deficiency and the anthropometric or metabolic derangements at the cut-off levels used. Results of ultra sound scans of abdomen were available in111 children and fatty liver grade 1 and above were noted in 63 children. However, there was no statistically significant association between vitamin D deficiency and fatty liver according to the available USS findings.

## Discussion

Several studies have reported the association between obesity and vitamin D deficiency worldwide [22]. There are no previous studies on the prevalence of vitamin D deficiency in children with obesity in Sri Lanka. It is noted that majority of the children included in the study sample were deficient (75.2%) in vitamin D levels while 21.4% were in the insufficient range. A statistically significant inverse relationship between vitamin D levels and fasting insulin, 2 h post glucose insulin, HOMA-IR and hs CRP, supporting the close relationship with vitamin D deficiency and insulin resistance.

We did look into the association of vitamin D levels with markers of adiposity (WC, SFT and fat mass) in addition to selecting the study sample according to BMI values. There was statistically significant negative correlation of vitamin D levels and SFT. However the negative correlation of WC and fat mass with vitamin D levels was not statistically significant. Studies have reported an inverse relationship of vitamin D levels with WC, which is the main marker of metabolic syndrome according to the International Diabetes Federation (IDF) criteria [16] and also SFT [23]. Metabolic syndrome in children aged ≥10 years can be diagnosed with abdominal obesity (based on WC) and the presence of two or more other clinical features (elevated triglycerides, low HDL-C, high blood pressure, increased plasma glucose) [15]. The association with other components of metabolic syndrome and vitamin D was not clear in other studies [24, 25] as well as in ours. Seeing an association with skin fold thickness and not with WC or fat mass could be due to several reasons. Since sample was selected based on high BMI, it is likely that all subjects had a high WC and fat mass as well and since there was limited variation seen in these two parameters, a significant correlation may not be seen. On the other hand, this may indicate that Vitamin D deficiency is more associated with subcutaneous fat deposition, which is supported by the findings of Didrikson et al. [26] who show that large amounts of vitamin D is stored in subcutaneous fat tissue. The mechanism of this storage and its significance however, needs to be explored further in future research studies. Furthermore, seeing an association with central skin fold thickness and not seeing an association with fat mass and waist circumference could be due to the effect of other confounding factors like diet, sun exposure etc. as well as fat mass and waist circumference represent visceral fat as well.

Animal studies have shown that vitamin D is associated with glucose mediated insulin secretion [27] which increases the expression of the insulin receptor and enhancing insulin-mediated glucose transport into tissue [28]. Several studies in adults have reported an association of vitamin D deficiency with insulin resistance and its improvement with supplementation [29, 30] and the results of our study also showed significant negative correlation of vitamin D levels with markers of insulin resistance(fasting insulin,2 h post glucose insulin and HOMA – IR). Furthermore vitamin D is known to have anti-inflammatory properties, which explains its deficiency resulting in development of type 2 diabetes [31]. CRP is an acute-phase protein, which rises in response to inflammation having a prognostic value in predicting the future risk of cardiovascular events and development of diabetes mellitus [32, 33]. The hs-CRP and vitamin D levels showed a statistically significant negative correlation in our study sample favoring the above fact.

Even though we observed a high prevalence of vitamin D deficiency and insufficiency in our study, high PTH levels were noted only in 11 (7.2%) and all of them had vitamin D levels in the deficient range. This is in keeping with the possible explanation that the activation of PTH axis in obesity happens with much lower levels of vitamin D compared to normal [34]. PTH and Vitamin D levels were negatively correlated. The Mean vitamin D level in the PTH-high group was significantly lower than that of the PTH-normal group (13.0 vs 17.6, *p* = 0.007). This is consistent with the physiological feedback mechanism of vitamin D on the parathyroid hormone secretion.

We have not looked into the levels of vitamin D in non-obese children as a comparison. Among the scarce data regarding the prevalence of vitamin D deficiency among Sri Lankan children, a study done on healthy 2–5 year old children (*n* = 340) from an urban area of western province showed prevalence of vitamin D deficiency (< 20 ng/ml) to be 34.7% [8]. A recent case control study done on vitamin D deficiency and its association with adiposity in primary school children aged 8–9 years in Sri Lanka showed that76.5% in children with high adiposity and 66.2% with normal adiposity among the boys, and 92.4% with high adiposity and 73.8% with normal adiposity among the girls had vitamin D deficiency (< 20 ng/ml), in keeping with our study findings [35]. A hospital based cross sectional study done on apparently healthy 1–5 year old children in India has shown a prevalence of 78% [36].

We have not assessed a detailed dietary history, physical activity and the amount of sun exposure in our participants, which can affect the levels of vitamin D apart from obesity. However, the exposure to sun in Sri Lanka (especially in the western province where all subjects came from)being high it could be assumed that the contribution made by sun exposure to vitamin D levels of the body could not be sufficient to provide adequate levels of vitamin D in obese individuals. The conduct of the study being confined to the months of October through to April, one could argue that the seasonality could have a bearing on results. However, Sri Lanka being a tropical country with sunlight and rainfall abundant throughout the year, it is unlikely that the seasonal variation could have affected the results [37]. Further studies are needed to identify the contribution of these modifiable factors on vitamin D deficiency.

Not having a control group (children with a normal BMI) in assessing the prevalence of vitamin D deficiency was a main limitation in our study. It would be useful to have national prevalence data, as there is growing evidence of the role of vitamin D in preventing the development of diabetes mellitus apart from its effect on Calcium homeostasis.

## Conclusion

The prevalence of vitamin D deficiency was very high among obese children participating in this study. Vitamin D levels show a significant association with insulin resistance and measures of adiposity. However, no statistically significant association was seen between vitamin D deficiency with fasting blood glucose and lipid profile.

## Data Availability

The data sets used and/or analyzed during the current study are available from the corresponding author on reasonable request.

## Abbreviations

ALT: Alanine aminotransferase
AST: Aspartate aminotransferase
BMI: Body mass index
FBG: Fasting blood glucose
HDL: High density lipoprotein
HOMA IR: Homeostatic model assessment of insulin resistance
Hs-CRP: High sensitivity C reactive protein
IDF: International Diabetes Federation
LDL: Low density lipoprotein
OGTT: Oral glucose tolerance test
PTH: Parathyroid hormone
RBG: Random blood glucose
SDS: Standard deviation score
SFT: Skin fold thickness
SPSS: Statistical Package for the Social Sciences
T2DM: Type 2 diabetes mellitus
USS: Ultra sound scan
WC: Waist circumference
